# Which urban communities are susceptible to COVID-19? An empirical study through the lens of community resilience

**DOI:** 10.1186/s12889-021-12419-8

**Published:** 2022-01-11

**Authors:** Chunyu Shi, Liao Liao, Huan Li, Zhenhua Su

**Affiliations:** 1 Discipline of Public Administration, School of Public Management, Zhejiang Gongshang University, Hangzhou, China; 2 School of Politics and Public Administration, China South Normal University, University City, Guangzhou, China; 3 Discipline of Land Resources Management, School of Public Management, Zhejiang Gongshang University, Hangzhou, China; 4 College of Media and International Culture, Zhejiang University, Hangzhou, China

**Keywords:** COVID-19, Pandemic-susceptible communities, Community resilience, Qualitative comparative analysis, China

## Abstract

**Background:**

After the lockdown of Wuhan on January 23, 2020, the government used community-based pandemic prevention and control as the core strategy to fight the pandemic, and explored a set of standardized community pandemic prevention measures that were uniformly implemented throughout the city. One month later, the city announced its first lists of “high-risk” communities and COVID-19-free communities. Under the standardized measures of pandemic prevention and mitigation, why some communities showed a high degree of resilience and effectively avoided escalation, while the situation spun out of control in other communities? This study investigated: 1) key factors that affect the effective response of urban communities to the pandemic, and 2) types of COVID-19 susceptible communities.

**Methods:**

This study employs the crisp-set qualitative comparative analysis method to explore the influencing variables and possible causal condition combination paths that affect community resilience during the pandemic outbreak. Relying on extreme-case approach, 26 high-risk communities and 14 COVID-19 free communities were selected as empirical research subjects from the lists announced by Wuhan government. The community resilience assessment framework that evaluates the communities’ capacity on pandemic prevention and mitigation covers four dimensions, namely spatial resilience, capital resilience, social resilience, and governance resilience, each dimension is measured by one to three variables.

**Results:**

The results of measuring the necessity of 7 single-condition variables found that the consistency index of “whether the physical structure of the community is favorable to virus transmission” reached 0.9, which constitutes a necessary condition for COVID-19 susceptible communities. By analyzing the seven condition configurations with high row coverage and unique coverage in the obtained complex solutions and intermediate solutions, we found that outbreaks are most likely to occur in communities populated by disadvantaged populations. However, if lacking spatial-, capital-, and governance resilience, middle-class and even wealthy communities could also become areas where COVID-19 spreads easily.

**Conclusions:**

Three types of communities namely vulnerable communities, alienated communities, and inefficient communities have lower risk resilience. Spatial resilience, rather than social resilience, constitutes the key influencing factor of COVID-19-susceptible communities, and the dual deficiencies of social resilience and governance resilience are the common features of these communities.

## Background

**Fig. 1:**
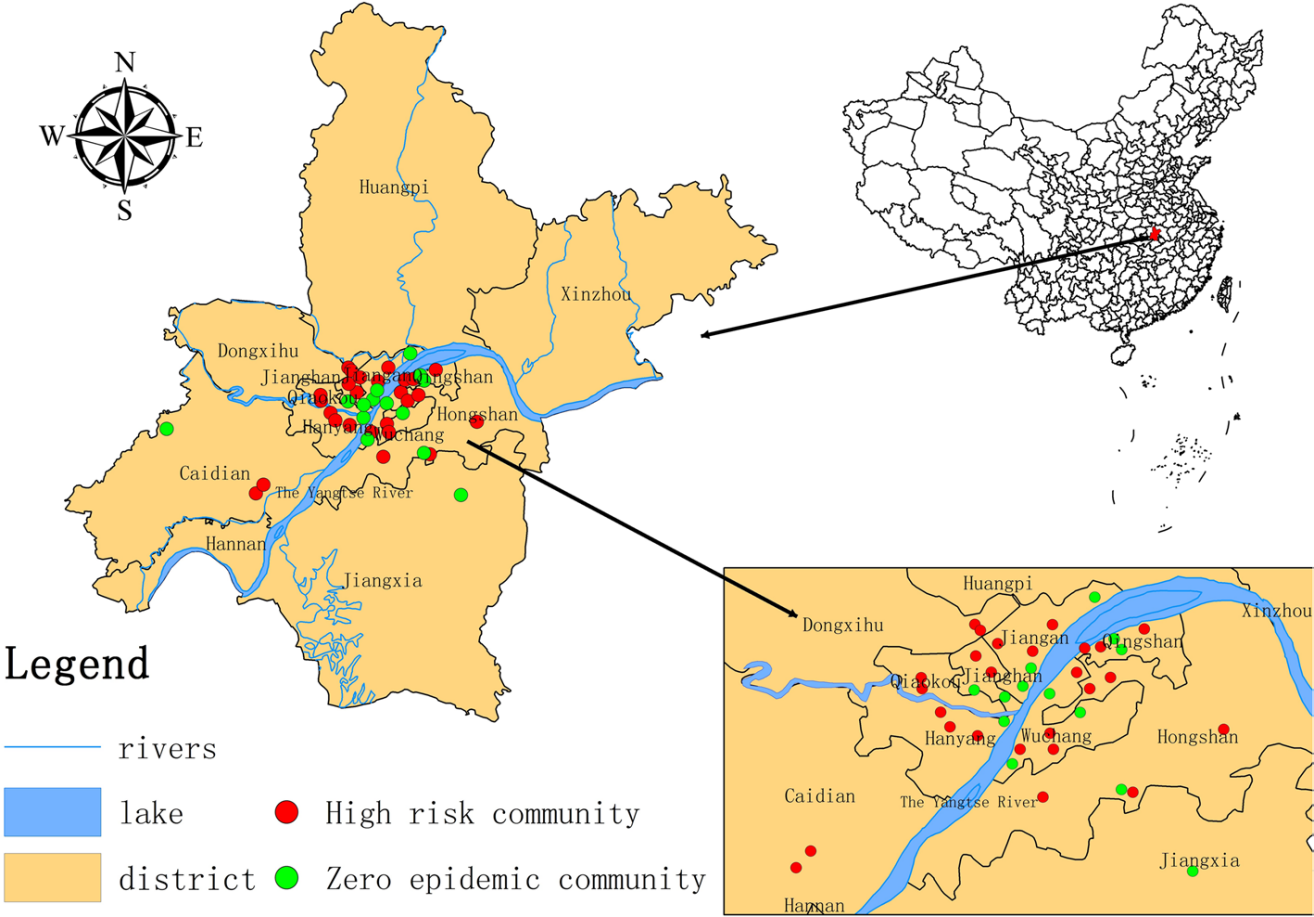
Map of communities included in this study

The global outbreak of COVID-19 has made prevention and mitigation of the pandemic an issue of urgent concern internationally [1]. The community is the basic functional defense unit of risk management [2] and its actions will significantly affect the overall level of disaster prevention and reduction in a city [3, 4]. However, faced with the threat of the pandemic, some communities showed a high degree of resilience and effectively avoided escalation, while the situation spun out of control in other communities. What are the factors that affect a community’s ability to respond effectively to a disaster? Which urban communities are vulnerable to COVID-19? To address these questions, this study selected 40 urban communities in Wuhan, China, as empirical research subjects (See Fig. 1). A community resilience assessment framework was developed and qualitative comparative analysis was applied to analyze the relationship between urban community resilience and the spread of COVID-19.

Wuhan is the capital of Hubei Province. The city is composed of 15 administrative districts and 1107 communities. Until 2020, Wuhan has a permanent resident population of 12,32 million, ranking the sixth-largest city in China [5]. In Wuhan, a community is composed by 3000 households, with populations over 10,000. The number of personnel allocated to the main management body of the community, namely resident committees, is varied from six to nine [6]. Besides the residents’ committee, each community has got a Party branch (general branch) composed of at least three Party members, and several sub-branches according to the number of Party members in the community. These Party branches play an important role in the daily governance of the community, and objectively bring important human resources to the governance of urban communities when the resident committees is seriously insufficient in this issue. Furthermore, in practice, Party members constitute the backbone and active participants in the process of community management. In addition to residents’ committees and Party branches, China’s community governance organizations also include property management agencies and homeowners’ autonomous organizations, namely homeowners’ committees. These four institutions constitute the four carriages of the community governance structure [7].

In the Chinese context, communities are the first state-backed organizations [8] and hence the implementers of the top-down policies initiated by the state [9]. Accordingly, as the communities are the link between state and society, they become important interlocutors for coproduction of effective disaster response [10]. After the lockdown of Wuhan on January 23, 2020, the government of the city adopted community-based pandemic prevention and control as the core strategy to fight the pandemic. Gradually, local governments explored a set of standardized community pandemic prevention measures that were uniformly implemented throughout the city. That is, a leading team for pandemic prevention was established at the community level with the secretary of the community Party branch as the team leader, the director of the residents’ committee as the deputy leader, and committee members as the team members. The leading team was in charge of the specific pandemic prevention work. Case reporting, pandemic investigation, time limits for case handling, procedures for pandemic prevention and control (isolation, disinfection, information, notification, pandemic tracking, etc.) were standardized in all communities.

On February 24, the Wuhan New Crown Pneumonia Pandemic Prevention and Control Headquarters announced the first list of 278 “high-risk” communities and residential blocks in Wuhan (more than 10 confirmed cases were considered as high-risk areas, involving 155 communities) [11]. On March 6, this office released the first list of COVID-19-free areas (no confirmed or suspected cases in the past 14 days), which included 79 communities and 2076 residential blocks [12]. According to the two lists, there were 16 high-risk communities and residential blocks in Jianghan District, the administrative district where the South China Seafood Market is located. Wuchang District and Qingshan District were the most severely affected, each with 45 high-risk communities. Surprisingly, neither of them is adjacent to the South China Seafood Market, where the pandemic first broke out, and the distance between the two districts is more than 10 km. These observations indicate that there is no direct geographic-distance relationship between the spread of the pandemic and the place where the pandemic was first discovered. The ability to prevent a pandemic is the key factor that determines whether a community is immune to the impact of the pandemic. Thus, we propose that communities with strong resilience are less vulnerable to the impact of the pandemic. By contrast, communities with poor resilience are more likely to fall victim to the pandemic.

### Literature review

Resilience is originally a concept from physics that refers to the ability of a substance or system to return to its original position after being displaced [13, 14]. In 1973, Canadian ecologist Holling introduced the concept into ecology to define the natural system’s ability to adapt to disasters [15]. Since then, the concept of resilience has been widely used in the field of urban studies [16], with emphasis on scientific issues, such as urban sustainable development [17] and urban disaster management. Improving urban resilience through community interventions has been seen as a promising paradigm for the promotion of disaster risk reduction [18]. Research aiming to frame the concept of community resilience is abundant, Patel et al. identified three types of definition [19]: 1) process definitions, resilience as an ongoing process or capacity of change and adaptation to community disaster; 2) absence of adverse effect definitions, resilience as an ability to maintain stable functioning; and 3) attributes definitions, resilience as a ‘range of attributes’, a broad collection of response related abilities. By emphasizing the adaptive abilities and attributes to reduce the impact of disasters, “the concept of ‘community resilience’ is almost invariably viewed as positive, being associated with increased local capacity, social support and resources, and decreasing risks, miscommunication and trauma [19].”

Like community resilience definition, there is no consensus as to how it should be measured. Depending on different conceptualization approaches and specific resilience domains to be assessed, measurement tools vary from quantitative methods relying on preexisting datasets, to qualitative and participatory framework using focus groups, interviews, and mixed approaches which combine quantitative and qualitative data [20]. Among the extensive assessment approaches on community resilience, the TOSE framework proposed by Bruneau et al. [21, 22] and the CDRF (community disaster resilience framework) constructed by Peacock et al. [23] have been widely recognized. Bruneau et al. regard the community as an organic system and believe that community resilience is composed of four dimensions: technical resilience, organizational resilience, social resilience, and economic resilience. In the CDRF approach, Peacock and others suggest that community resilience is mainly determined by the community’s resources and endowments. These endowments include the stock of social capital, economic capital, physical capital, human capital, and natural capital. A community with a larger capital stock has greater resilience to external shocks [24, 25]. The different measurement strategies emphasized by these authors show that the assessment framework needs to take account of the multidimensional nature of community resilience [13], including those related to the natural and physical resources of the community environment, social structures (community networks), economic elements (employment and financial savings), demographical structure (age, education, physical conditions), and institutional aspects such as community organizations, and governance structures [26].

Developing a proper and adequate community resilience measurement framework remains a complex challenge. Clark-Ginsberg et al. point out that “community resilience measurement is less about developing measures that can satisfy all design criteria and more about minimizing the negative elements of measurement choices and maximizing the positive” [27], and that it should put efforts to balance measurement demands including validity, reliability, ease of use, and utility. South et al. suggest there is a need to contextualize information on community resilience by taking into account differences between communities and the specific disaster they undergo. Similarly, other scholars insist that measurement strategies should be based on specific communities and scenarios [28].

Despite their important theoretical and empirical contributions, the current literature presents some limitations. First, studies on community resilience mostly focus on the community’s ability to adapt to natural disaster risks in the context of climate change [25], research on public health remains insufficient [29]. The COVID-19 pandemic has brought “resilience” back to the research perspective of community public health risks [30], but researchers are mainly interested in discussing the community’s resilience to the pandemic from the perspective of human capital, social capital [31], and (risk) governance systems [32]. This leaves the literature without an in-depth analysis of spatial elements, which also influence significantly the spread of the pandemic. Second, there is also a lack of concern about the context which “is the key to understanding community-level vulnerabilities” [28], the institutional and socio-cultural context could strongly influence community resilience that requires more specialization in the choice of measurement strategies [28]. Third, although there exists a variety of assessment methods, most studies tend to be quantitative studies based on complex measurement indicators, the different factors that affect community resilience are often regarded as equally important [27], leaving insufficient knowledge about the main factors that determine the community resilience and the ways they interrelate [28, 33].

To complete the theoretical and methodological deficiencies of existing research, this study adopted a qualitative comparative method to investigate the influencing factors and their dynamic interactions that affect community resilience during the pandemic outbreak. Through the analysis of 40 urban communities in Wuhan, we identified key factors and common features that determine communities’ capacity for pandemic prevention and mitigation, as well as types of communities susceptible to the COVID-19 pandemic. This study aims to provide theoretical inspiration for urban communities to respond to the same type of public health risk.

## Methods

### Qualitative comparative analysis

Qualitative comparative analysis (QCA) is a research method oriented toward small and medium sample case studies (10–40). Through a comparative analysis of causal conditions in multiple cases, it seeks to reveal multiple conjunctural causation relationships between conditional variables and outcome variables [34]. Multiple configurational causality implies three elements [35]. First, it is a combination of several different conditions (independent or “explanatory” variables) that eventually causes an outcome (dependent variable, or phenomenon to be explained). Second, causality is context- and conjuncture-sensitive, depending on the context, a given condition may have different – or even opposite – effects on the outcome, different combinations of conditions thus might produce multi finality or conjunctural causation. Third, multiple configurational causality indicates phenomena of equifinality, which means different causal paths (combinations of conditions) might lead to the same outcome. By using QCA, researchers are suggested to “determine the number and character of the different causal models that exist among comparable cases” [36], but not to identify a single causal model that fits the data best [35].

At present, QCA has developed three research methods: crisp-set qualitative comparative analysis (csQCA), fuzzy-set qualitative comparative analysis (fsQCA), and multi-value qualitative comparative analysis (mvQCA). Among them, crisp-set QCA is the most widely used method by researchers [37–39]. Its basic principle is to set the condition variable and result variable as dichotomous variables, namely “yes” or “no” or “true” or “false,” with values of 1 and 0, respectively. The groundwork of csQCA is to assume causal complexity and then analyze that complexity [36]. To operate a csQCA, a researcher should first elaborate a data table namely a truth table, in which each case is interpreted by a specific combination of condition variables and an outcome variable. The software then calculates the truth table and produces a list of causal configurations (2^i^ configurations in total, i = number of condition variables). A specific configuration is the combination of some condition variables and an outcome variable, which might correspond to several observed cases. In this sense, QCA is useful for data exploration and case interpretation in a synthetic way, as a relevant tool for typology-building [35]. It can also be useful for theory-testing and allows one to elaborate on new theories or propositions [40].

To examine whether there is a necessary and sufficient relationship between a given condition variable and an outcome variable, one should measure the consistency and coverage of a single condition variable in the overall sample cases.

The univariate necessity and adequacy analysis are assessed by the consistency index. The formula for calculating the consistency index is as follows:

$$\mathrm{Consistency}\;\left(\mathrm{Xi}\leq\mathrm{Yi}\right)=\sum\left[\min\left(\mathrm{Xi},\;\mathrm{Yi}\right)\right]/\sum\mathrm{Xi}$$

If variable X (single conditional variable or combination of conditional variables) is a sufficient condition for Y (outcome variable), the consistency index should be greater than 0.8 [41]. At the same time, the consistency index (Yi ≤ Xi) can be used to assess whether X is a necessary condition for Y. If it is greater than 0.9, then X can be considered as a necessary condition for Y [41]. After completing the assessment of sufficient or necessary conditions, the coverage index can be used to assess the interpretation strength of the condition (or combination) X to the outcome Y. The coverage rate formula is simplified as follows:

$$\mathrm{Coverage}\left(\mathrm{Xi}\leq\mathrm{Yi}\right)=\sum\left[\min\left(\mathrm{Xi},\;\mathrm{Yi}\right)\right]/\sum\mathrm{Yi}$$

This indicator describes how well the condition (or combination) X interprets the outcome Y. The larger the value of the coverage index, the stronger the explanatory power of X to Y.

This study chooses csQCA as the analysis method. The reason is as follows: First of all, the boundary of the condition variables and outcome variables involved in the study is relatively clear, and can be divided into two categories by “true” or “false”. Second, with the combination comparison and calculation of multiple concurrent conditions in multiple cases, a csQCA study can help identify the necessary conditions and sufficient conditions combination paths that lead to the emergence of COVID-19-susceptible communities. This can partly compensate for the limitations of current quantitative or qualitative researches on this issue. In addition, through the analysis of multiple concurrent configurations between condition variables and outcome variables, qualitative comparative research can also help reveal the complex dynamic interactions between factors that influence community resilience.

### Community resilience assessment framework and variables’ coding

This study integrated and improved the assessment frameworks put forward by Bruneau et al. and Peacock et al., and developed a community resilience assessment framework covering the four dimensions of spatial resilience, capital resilience, social resilience, and governance resilience. Each dimension is measured by 1–3 variables. As the total number of variables in a QCA is usually limited from four to seven [42], the study could not take into account every potential influencing factor, therefore we use synthetical qualitative variables to assess community resilience, a variable is the composite of several qualitative and quantitative factors, which allows for a holistic picture of a specific local condition [35]. Their ability to represent complexity makes them useful for measuring multidimensions of community resilience simply and comparably. The specific connotation of the four dimensions as well as of their measurement variables are described below. We remind that the Chinese communities’ specific characteristics have been taken into consideration when we defined the condition variables.

#### Spatial resilience

This term refers to the resilience of community physical space, building environments, and public infrastructure, such as community space planning, public facilities, characteristics of buildings and residential houses, and disaster prevention facilities [22, 23, 43] Communities with sufficient redundancy in space and strong robustness in facilities are resilient [44]. The spatial resilience in this study was measured by the conditional variable “whether the physical structure of the community is favorable to virus transmission”. According to findings revealed by existing studies, there are two main types of communities that have been identified as areas susceptible to pandemics. The first type is communities characterized by high-rise residential buildings [45]. According to a WHO report on the SARS pandemic, high-rise residential buildings are favorable to the virus spreading from one room to another room via the high-drop sewer- and plumbing network [46]. In reality, not only the sewage systems, facilities such as elevators and shared ventilation shafts also lead to high-rise residential buildings becoming hotbeds for the spread of the virus. The second type of high-risk area is the slum [47], known in Chinese as the “old-broken-small” community. Common space and living space are often very narrow in these communities, the lighting and ventilation conditions of the buildings are poor, and the public health facilities are usually outdated or even deficient [48]. The poor spatial conditions enable viruses to spread easily among the population with their high density and frequent encounters [49]. A value of 1 was assigned to cases belonging to communities characterized by high-rise buildings or old-broken-small housing, and a value of 0 was assigned to other cases.

#### Capital resilience

Communities incorporate a stock of human- and economic capital that defines their capital resilience. This resilience is defined by the community population characteristics, such as education and skills, the employment rate, and the average income level of families [22, 23]. Communities with abundant human- and economic capital can absorb and adapt to the impact of disasters quickly [23]. In this study, capital resilience was measured by the condition variable “whether it is a community populated by vulnerable groups”. Vulnerable groups are often risk-susceptible because the human- and economic capital that they can mobilize is limited and their ability to respond to risks is poor [50]. Certain communities tend to be populated with a large proportion of poor and disadvantaged people. Their common characteristics include high proportions of elderly, migrants, unemployed, people with disabilities, and individuals living on subsistence allowances. The coding was based on the self-positioning of the community in official documents, and the study assigned a value of 1 to communities considered as vulnerable by their administrators, and a value of 0 to others.

#### Social resilience

It involves the quality and stock of a community’s social capital, which is reflected through the number of community social organizations, the relationship between residents, and their participation in community affairs [22, 23]. Resilient communities have more social capital stock and stronger collective action effectiveness [51]. In this study, social resilience was measured by conditional variables “whether it is a young community” and “whether communities have few social organizations”. (1) Young community members have strong structural heterogeneity, residents are strangers to each other, they are atomized individuals or families, with a low degree of interpersonal interaction, mutual trust and cooperation are rare between them, and the community cohesion is weak. The authority and mobilization capacity of community official organizations, such as residents’ committees, are often limited; therefore, young communities’ social capital is usually weak, and collective action capacities are poor. The ability of communities to respond to risk disasters is therefore low. Young communities in China mainly are of two types: high-rise commercial housing residential communities [52] and resettlement housing communities. They are new communities created by the government following the demolition and restructuring movement since 2000 [53]. Residents often come from different communities and have a low degree of familiarity and interaction with each other; therefore, the community lacks cohesion. The cases belonging to the above two types of communities are regarded as young communities and assigned a value of 1. Otherwise, they are assigned a value of 0. (2). The number of community social organizations is one of the main indicators to measure the stock of community social capital. It is generally believed that social organizations are the main platform for community residents to communicate and interact, and help residents form a social network with a certain cooperative and mutual-trust relationship [23]. The larger the number of social organizations, the stronger the resilience of the community, and vice versa. A value of 1 was assigned to cases in which the number of social organizations was lower than the median of 4, and a value of 0 was assigned to the contrary.

#### Governance resilience

It refers to the level of a community’s governance capabilities and the abundance of governance resources. It is reflected through the completeness of the community governance structure, the community decision-making and implementation capabilities, and the adequacy of human and institutional resources on community governance [22, 33, 54]. Governance resilience is measured by the condition variables “whether the number of Party committee’s branches is small”, “whether it is an advanced/demonstrative community”, and “whether residents have complained to the community administrators due to their poor implementation of anti-pandemic measures”. (1) As the Party branch incarnates the leadership and constitutes the backbone of community governance, the construction of the Party Committee’s branches affects the degree of community organization and the synergy of various types of institutions and organizations. When the Party branches are efficient, and the more Party members there are, the higher the community governance capability, and the stronger its resilience. In this study, the community cases with the number of Party branches below the median of 3 were assigned a value of 1, otherwise, a value of 0. (2) Advanced/demonstrative communities reflect that community administrators have a high sense of responsibility and a high community management ability, community residents have a relatively high degree of cooperation and participation in community affairs, and the community has a certain degree of cohesion. Its defense and adaptability to disasters are therefore often relatively strong. Cases that have not been rated as advanced or demonstrative communities will be assigned a value of 1, and other cases will be assigned a value of 0. (3) Because all communities in Wuhan are required to adopt standardized anti-pandemic measures, it is difficult to assess the risk management capability of the community by the indicator “whether or not anti-pandemic measures are taken,” but it is possible to measure the effectiveness of the community’s anti-pandemic measures and their anti-pandemic capabilities by whether the community administrators have received complaints from residents. This study assigns a value of 1 to the community being complained about and 0 to those not being complained about.

Lastly, as for the outcome variable “Whether it is a COVID-19 susceptible community”, we assign a value of 1 to high-risk communities and 0 to COVID-19 free communities (see Table 1 below).

**Table 1:** Variables and coding rules

Resilience Dimensions	Variables	Coding rules
Spatial resilience	(1)whether the physical structure of the community is favorable to virus transmission(physical structure)	“true”, 1; “false”, 0
Capital resilience	(2)whether it is a community populated by vulnerable groups (vulnerable groups)	“true”, 1; “false”, 0
Social resilience	(3)whether it is a young community (young community)	“true”, 1; “false”, 0
	(4)whether communities have few social organizations (social organizations)	“true”, 1; “false”, 0
Governance resilience	(5) whether the number of Party committee branches is small (Party committee)	“true”, 1; “false”, 0
	(6)whether it is a non-advanced/demonstrative community (non-advanced community)	“true”, 1; “false”, 0
	(7)whether residents have complained to the community administrators due to their poor implementation of anti-pandemic measures (residents’ complaints)	“true”, 1; “false”, 0
Outcomes	Whether it is a COVID-19 susceptible community	high-risk community, 1; COVID-19 free community, 0

### Case sample selection and data processing

As the purpose of this study is to investigate which and why some communities are susceptible to COVID-19, we use an extreme-case approach to select case samples. According to Mills et al., “the extreme cases approach is employed when the purpose is to try to highlight the most unusual variation in the phenomena under investigation” [55]. It is a case sampling strategy frequently used in risk assessment researches to understand in depth a particular risk phenomenon [56]. The case selection in this article is based on the following principles: (1) the severity of the community pandemic, that is, communities with the most infections in the same district are selected as the research cases; (2) the principle of appropriate proportion. The number of cases is arranged according to the severity of the pandemic in the district. The number of cases in districts severely hit, such as Wuchang and Qingshan, is greater than that in other districts; (3) the availability and completeness of community data. Cases in which complete data are difficult to obtain are excluded because they cannot be compared with other cases.

Research data mainly are originally collected from government information disclosure via the Internet and social networks. The data related to the cases are taken from the China Community website(www.cncn.org.cn). The data for the condition variable “whether community administrators have received complaints due to poor implementation of anti-pandemic measures” are taken from the “Leadership Message Board” of People’s Daily Online (liuyan.people.com.cn) and Weibo (weibo.com). Some information that is difficult to verify through the Internet, such as whether the physical spatial characteristics of individual communities are high-rise buildings or old-broken-small housing, was verified by residents in Wuhan through telephone interviews.

Based on the above sample selection principles and data sources, this study selected 40 communities in Wuhan as research cases. Among them, 26 cases were from the first “high-risk” community list published by Wuhan COVID-19 Prevention and Control Headquarter on February 24, 2020, and the other 14 cases were from the first “COVID-19 free” community list published by the same office on March 6, 2020. From January 23, 2020, Wuhan city was locked down, and the next day Hubei Provincial government launched the level-I response to the public health emergency, the pandemic prevention and control measures in Wuhan had been carried out for more than a month, which is long enough to reflect the differences in resilience of different communities to respond and adapt to the pandemic.

According to the variable setting and assignment rules, 40 cases were coded, and the resulting truth table is as follows (Table 2).

**Table 2:** Truth table exhibiting the coding of the 40 cases

Variables	Physical structure	Vulnerable groups	Young community	Social organizations	Party committee	Non-advanced community	Residents’ complaints	Outcomes
Cases								
C1	1	1	1	1	1	1	1	1
C2	1	1	1	0	1	1	0	1
C3	1	0	1	0	1	1	1	1
C4	1	1	0	1	1	1	0	1
C5	1	1	1	1	1	1	1	1
C6	0	0	1	1	1	1	1	1
C7	1	1	1	0	0	1	1	1
C8	1	1	1	0	1	1	1	1
C9	1	1	1	0	0	0	1	1
C10	1	0	1	0	0	1	0	1
C11	1	1	1	1	1	1	1	1
C12	1	1	0	1	0	0	1	1
C13	0	1	1	1	1	1	1	1
C14	0	0	1	1	1	1	1	1
C15	1	1	1	1	1	1	0	1
C16	1	1	1	0	0	1	0	1
C17	1	0	1	0	0	1	0	1
C18	1	1	1	0	0	1	0	1
C19	1	0	1	0	0	1	0	1
C20	1	0	1	0	0	0	1	1
C21	1	0	1	1	0	1	0	1
C22	1	1	1	1	0	1	0	1
C23	1	0	1	0	1	1	1	1
C24	1	0	0	1	0	1	1	1
C25	1	0	1	0	1	0	0	1
C26	0	1	1	1	1	1	0	1
C27	0	0	0	1	0	0	0	0
C28	1	1	0	0	0	1	0	0
C29	0	0	0	0	0	1	0	0
C30	0	1	0	0	0	0	0	0
C31	0	0	0	1	0	1	0	0
C32	0	1	0	0	0	1	0	0
C33	1	1	0	0	0	0	0	0
C34	0	0	0	0	0	0	0	0
C35	0	0	0	0	0	0	0	0
C36	0	1	0	0	0	1	0	0
C37	0	1	0	0	0	0	0	0
C38	0	0	0	0	0	1	0	0
C39	0	0	0	0	0	1	0	0
C40	0	1	0	1	1	0	0	0

## Results

### Analysis of the necessary conditions for COVID-19-susceptible communities

fsqca3.0 software [57] was used to analyze the case truth table so as to obtain the results of the consistency and the coverage of each condition variable.

If the condition variable X is a necessary condition for the outcome variable Y, the consistency index of the variable should be > = 0.9. As shown in Table 3, the results of measuring the necessity of 7 single-condition variables found that the consistency index of “whether the physical structure of the community is favorable to virus transmission” reached 0.9, which constitutes a necessary condition for “COVID-19 susceptible communities,” and its case coverage rate is 0.846, indicating that the condition variable has strong explanatory power. The resilience dimension corresponding to this variable, namely spatial resilience, has therefore become a key influence dimension of the community’s ability to prevent and control pandemics.

**Table 3:** Necessary and sufficient conditions for COVID-19 susceptible communities

Variables	Outcomes	
	Consistency	Coverage
Physical structure	0.916667	0.846154
Vulnerable groups	0.750000	0.576923
Young community	0.833333	1.000000
Social organizations	0.500000	0.857143
Party committee	0.666667	1.000000
Non-advanced community	0.833333	0.588235
Residents’ complaints	0.750000	1.000000

The consistency of the condition variable “young community” exceeds 0.8, and the coverage rate reaches 1.0. This finding is indicative that, although this variable does not constitute a necessary condition for the emergence of COVID-19-susceptible communities, it is a sufficient condition, having an important impact on community resilience that cannot be ignored.

### Analysis of sufficient condition configurations

Using fsqca3.0 software to analyze the case truth table, three sufficient condition configuration paths were obtained: complex solutions, parsimonious solutions, and intermediate solutions(Table 4). As the parsimonious solutions are too simple to reveal the complexity of the causality, researchers usually focus their analysis on complex solutions or intermediate solutions. This study thereby analyzed the seven condition configurations with relatively high row coverage and unique coverage in the obtained complex solutions and intermediate solutions, their consistency and result coverage are both 1 (Table 4), which indicate that the explanatory power of these seven configurations to COVID-19 susceptible communities can reach 100%.

**Table 4:** Sufficient condition configurations for COVID-19 susceptible communities

Solutions	Configurations	Row coverage	Unique coverage	Consistency
Complex solutions	Physical structure * Vulnerable groups * Young community * Social organizations * Party committee * Non-advanced community	0.166667	0.0833333	1
	Physical structure * Young community * Party committee *Non-advanced community* Residents’ complaints	0.166667	0.0833333	1
	Physical structure * Vulnerable groups * Young community *Party committee * Non-advanced community*Residents’ complaints	0.333333	0.25	1
	Physical structure * Vulnerable groups * Social organizations *Party committee *Non-advanced community	0.0833333	0.0833333	1
	Coverage	1.000000		
Intermediate solutions	Physical structure * Vulnerable groups * Young community *Residents’ complaints	0.166667	0.166667	1
	Physical structure * Young community * Non-advanced community	0.0833333	0.0833333	1
	Young community * Social organizations * Party committee * Non-advanced community *Residents’ complaints	0.0833333	0.0833333	1
	Coverage	1.000000		
parsimonious solutions	Young community	0.833333	0.583333	1
	Vulnerable groups * Social organizations	0.416667	0.166667	1
	Coverage	1.00000		

Configuration 1: Physical structure * Vulnerable groups * Young community * Social organizations * Party committee * Non-advanced community. The community lacks spatial resilience. It is characterized by high-rise buildings or old-broken-small housing. Second, the community lacks capital resilience. It is a community populated by disadvantaged groups. Third, the community lacks social resilience and is a young community with few social organizations. Fourth, the community lacks governance resilience, has few Party branches, and it is also a non-advanced/demonstration community.

Configuration 2: Physical structure * Young community * Party committee *Non-advanced community* Residents’ complaints. The community lacks spatial resilience. It is characterized by high-rise buildings or old-broken-small housing. Second, the community lacks social resilience. It is a young community. Third, the community lacks governance resilience. There are few Party branches. It is a non-advanced/demonstrative community and has been the subject of complaints due to the ineffective implementation of anti-pandemic measures.

Configuration 3: Physical structure * Vulnerable groups * Young community *Party committee * Non-advanced community*Residents’ complaints. The community lacks spatial resilience. It is characterized by high-rise buildings or old-broken-small housing. Second, the community lacks capital resilience and is a community populated by disadvantaged groups. Third, the community lacks social resilience and is a young community. Fourth, the community lacks governance resilience. There are few Party branches, it is a non-advanced/demonstrative community, and it has been the subject of complaint due to ineffective implementation of anti-pandemic measures.

Configuration 4: Physical structure * Vulnerable groups * Social organizations *Party committee *Non-advanced community. The community lacks spatial resilience. It is characterized by high-rise buildings or old-broken-small housing. Second, the community lacks capital resilience. It is a community populated by disadvantaged groups. Third, the community lacks social resilience. The number of social organizations is small. Fourth, the community lacks governance resilience. There are few Party branches, and it is also a non-advanced/demonstrative community.

Configuration 5: Physical structure * Vulnerable groups * Young community *Residents’ complaints. The community lacks spatial resilience. It is characterized by high-rise buildings or old-broken-small housing. Second, the community lacks capital resilience and is a community populated by disadvantaged groups. Third, the community lacks social resilience. It is a young community. Fourth, the community lacks governance resilience and has been the subject of complaint due to ineffective implementation of anti-pandemic measures.

Configuration 6: Physical structure * Young community * Non-advanced community. The community lacks spatial resilience. It is characterized by high-rise buildings or old-broken-small housing. Second, the community lacks social resilience and is a young community. Third, the community lacks governance resilience and is not an advanced/demonstrative community.

Configuration 7: Young community * Social organizations * Party committee * Non-advanced community *Residents’ complaints. The community lacks social resilience. It is a young community, and the number of social organizations is small. Second, the community lacks governance resilience, with few Party branches. It is also a non-advanced/demonstrative community and has been the subject of complaint due to the ineffective implementation of anti-pandemic measures.

The seven configurations illustrate different situations leading to communities vulnerable to COVID-19. According to their vulnerability in different dimensions of resilience, the seven configurations could be classified into three categories: category 1 is overall vulnerable in four dimensions of resilience (configurations 1, 3, 4, and 5); category 2(configurations 2 and 6) is sufficient in capital resilience but vulnerable in spatial, social, and governance resilience; category 3(configuration 7) is sufficient in spatial and capital resilience, but vulnerable in social and governance resilience.

Among the seven configurations, six of the configurations are related to insufficient spatial resilience, four of the configurations are related to insufficient capital resilience. Spatial resilience thereby is the main factor that influences the degree of community resilience, the results confirm the findings of the above analysis of the necessary conditions for COVID-19 susceptible communities. Lastly, insufficient social resilience and governance resilience appear in all of the seven configurations, it’s the common feature of all COVID-19-susceptible communities.

### Typology of COVID-19-susceptible communities

The above seven condition combination paths involve three types of COVID-19-susceptible communities. This article names them vulnerable communities (configurations 1, 3, 4, and 5), alienated communities (configurations 2 and 6), and inefficient communities (configuration 7). See Table 5 below.

**Table 5:** Typology of COVID-19-susceptible communities

Typology	Spatial resilience	Capital Resilience	Social resilience	Governance resilience	Path	Case sample	Proportion (100%)
Vulnerable community	●	●	●	●	Configuration 1, 3, 4, 5	C1,C2,C4,C5,C7,C8,C9,C11,C12,C13,C15,C16,C18,C22,C26	57.7%
Alienated community	●		●	●	Configuration 2,6	C3,C10,C17,C19,C20,C21,C23,C24,C25	34.6%
Inefficient community			●	●	Configuration 7	C6,C14	7.7%

### Vulnerable communities

Vulnerable communities covered 57.7% of susceptible community cases. Because of its overall weakening characteristics in the four dimensions of spatial resilience, capital resilience, social resilience, and governance resilience, vulnerability is its core feature, so this type of community is named a vulnerable community. Its condition combination is configuration 1, configuration 3, configuration 4, and configuration 5.

First, in terms of spatial features, vulnerable communities fall into two categories. One is characterized by high-rise buildings and usually consists of social housing (such as Case 1), resettlement housing (such as Case 5), or urban villages (such as Case 2, Case 11). The other involves large non-gated old communities with poor common facilities and living conditions, namely old-broken-small communities. Second, in terms of human- and economic capital, vulnerable communities are urban areas populated by disadvantaged groups. The elderly, disabled, low-income households, and floating population account for a large proportion of the total community population. Third, the social capital of vulnerable communities is relatively weak. They are usually communities established after the year 2000 and belong to young communities. Residents come from different parts of the country, and their relationship networks and social bonds are weak. The apparent exceptions, such as cases 4 and 12, where the communities were established early on, however, share the common trait that they have only got a few social organizations. Therefore, they lack the channels and platforms for enhancing communication and cooperation among residents. Finally, vulnerable communities also belong to communities with low governance efficiency. These communities often lack governance resources. Except for the establishment of a general Party branch at the community level, there is no other Party branch, and community Party members have not been effectively organized and mobilized. This means these communities lack effective leadership organizations and active participants. In these communities, administrative members often receive complaints from residents in terms of competence and responsibility. Therefore, vulnerable communities lack robust resilience to effectively meet the needs of community residents for pandemic prevention and control.

After being designated as high-risk communities, vulnerable communities mainly rely on the support and help of external resources to overcome the pandemic. During the period of COVID-19, the general practice of Wuhan City was to support high-risk communities one-on-one by higher-level official departments, establishing temporary Party branches that played the role of leadership, sending extra Party members and government employees to augment the community’s pandemic response personnel, relying on the authority of higher-level departments and Party organizations to mobilize community residents and organizations to work together to fight the pandemic.

### Alienated communities

The main difference between alienated communities and vulnerable communities is that the former ones are not areas populated by vulnerable groups. The residents’ socio-economic status and human capital are in the middle or even the upper level of society. The reason why they are named alienated communities is that these communities are mostly young communities with a short history of establishment in which residents are strangers to each other, that is, alienated. Its condition combinations are configurations 2 and 6. The alienated communities cover 34.6% of COVID-19-susceptible community cases.

The vulnerability of alienated communities is mainly reflected in three dimensions: spatial resilience, social resilience, and governance resilience. They are usually new communities built around the real estate economy after the rise of the commercial housing market. In terms of housing type, they usually involve high-rise modern buildings. The residents are similar in social and economic status, that is, the middle- or upper class. However, these communities are often loosely organized communities, with few social organizations and low participation in community affairs. Party branch building is imperfect. As it is difficult for the homeowners to establish an autonomy committee, the property management agency thereby lacks supervision, and its conflicts with the homeowners are numerous. Community residents’ committees often find it difficult to gain the trust and cooperation of residents because of their bureaucratism in daily work. Due to their high socioeconomic status, community residents have a relatively strong awareness of rights, and they often put higher service requirements on community administrators. Additionally, the existing property management agencies and residents’ committees cannot effectively meet the expectations of residents. As a result, the administrators of this type of community are often vulnerable to complaints and resistance from residents during the pandemic. In short, despite the strong capital resilience of alienated communities, the lack of resilience to the pandemic in the design and planning of physical residential space, as well as insufficient social resilience and governance resilience, leaves this type of community vulnerable to COVID-19.

### Inefficient communities

Inefficient communities do not involve high-rise buildings or old-broken-small housing, nor are they inhabited by disadvantaged groups, but are communities lacking both social resilience and governance resilience. Because of their low collective action efficiency and governance efficiency, they are called “inefficient communities.” Their condition combination is configuration 7. Case 6 and Case 14 in this study belong to this type of community, covering 7.7% of risk-susceptible community cases.

Case 6 is a good illustration of inefficient communities. It involves a new community located in Qingshan District, Wuhan, established in 2002. According to the community’s information registered on the “China Community Network”: “The community covers an area of 141,700 square meters, with a total construction area of 222,000 square meters and a green area of 2.27 hectares. The community is surrounded by trees and has a beautiful environment. It is composed of five independent courtyards. There are 37 residential buildings, 160 unit doors, 2,289 households with 7,819 people, including 4 low-income households, 46 disabled people, and 243 elderly living alone.” In terms of physical space characteristics, the community has a low floor area ratio and a high greening rate. It is characterized by neither high-rise buildings nor old-broken-small housing, so it has strong spatial resilience. In terms of its capital resilience, the population of disadvantaged groups accounted for approximately 3.7% of the total population of the community. It is not a community populated by disadvantaged groups. It has strong resilience in human capital and economic capital. However, the community was established in 2000, it is a young community. The number of registered social organizations is only 3, which is lower than the average of 40 community cases. The community has only one Party branch composed of three Party members. The number of Party members is limited. The community has not received any reward from high-level governments. Moreover, during the pandemic, the community administrators received complaints from residents for not being “serious and responsible” (e.g., the building was not disinfected from top to bottom, and the infected person was allowed to isolate at home when he should have been sent to the hospital). The main reason for inefficient communities to become pandemic-susceptible communities is their weak social resilience and governance resilience; therefore it is difficult for them to resist the spread of the virus during the outbreak.

## Discussion: the key factor and common features of COVID-19 susceptible communities

The results of analysis of necessary conditions of the seven variables revealed that spatial resilience is the key influencing factor of COVID-19 susceptible communities, and the seven condition configurations showed that the dual deficiencies of social resilience and governance resilience are the common features of these communities. These issues are further discussed below.

### The key factor of COVID-19 susceptible communities

#### Community spatial resilience as the key factor

From the aforementioned univariate necessity analysis, it can be seen that insufficient spatial resilience constitutes the key factor of pandemic-susceptible communities. Among the 26 high-risk community cases, 22 cases lack spatial resilience, with a case coverage rate of 84.6%. Two types of communities are characterized by insufficient spatial resilience: First, residential communities are dominated by large-scale high-rise buildings, with high population density and frequent intercrossing between residents. The vertically enclosed shared facilities of high-rise buildings, such as elevators, ventilation facilities, and sewage facilities, can easily cause the virus to spread in the same building space. The second is the “old-broken-small” communities. Residential buildings usually have poor ventilation and lighting conditions, lack of basic sanitation facilities (it is common for many people to share toilets and kitchens), and poor environmental sanitation [58] making it easier for the virus to remain in these spaces and spread among the high-density population.

#### The role of community capital resilience

Among the 26 high-risk community cases, a total of 15 communities involved disadvantaged groups inhabited areas, accounting for 57.7%, and 13 of the latter lacked spatial resilience. However, among the 14 COVID-19-free cases, there were also 7 communities inhabited by disadvantaged groups. Among them, two communities lacked spatial resilience, but had strong social resilience and governance resilience; the other 5 communities had strong spatial resilience, social resilience, and governance resilience. The above data show that the key reason why communities inhabited by disadvantaged groups have become pandemic-susceptible communities may be that these groups often live in communities that lack spatial resilience, not their weak human and economic capital resilience. As long as communities lacking capital resilience have sufficient social resilience and governance resilience, they can also be protected from the impact of the pandemic.

If we closely examine the 14 cases of COVID-19-free communities, it can be found that communities inhabited by disadvantaged groups with sufficient social resilience and governance resilience are all old neighborhood communities with numerous social organizations. In this type of community, residents are relatively homogeneous in member structure and status. Most of the residents have known each other for decades, they have relatively stable and continuous social networks, and there exist mutual trust and cooperation relationships with each other. Traditional authority and administrative organizations, such as Party branches and residents’ committees, are relatively efficient, and they enjoy a high degree of authority and recognition among residents, so they have strong mobilization capabilities to enforce specific measures. The existing endowments and resources of the community can be converted into effective collective action efficiency and governance efficiency at the critical moment of disaster, providing resilience for efficient disaster response.

### Insufficient social resilience and governance resilience as common features of COVID-19 susceptible communities

Although each of the three types of COVID-19 susceptible communities has its unique characteristics, they share common features with a lack of social resilience and governance resilience.

Firstly, communities susceptible to COVID-19 are often communities low on organization and resident participation, in other words, lacking social resilience. In these communities, residents have not formed cooperative relationships based on mutual trust. Instead, the residents are unlikely to provide mutual assistance in the face of disasters, and mainly rely on the residents’ committee, property management agencies, and higher-level governments to fight the pandemic,

It is worth discussing that the number of social organizations is usually regarded as an important indicator to measure the stock of social capital in a community [22, 23]. Can all types of social organizations be converted into high-quality social capital? In our research cases, we found that the registered social organizations are mainly interest-oriented or service-oriented organizations, and political participation-oriented social organizations are rare. During the COVID-19 pandemic, community social organizations were generally absent from participation, while the main force to fight the pandemic was official organizations and Party members. For instance, in the Baibuting community in Jiang’an District, the community requires “organization to be established within 100 steps and residents’ participation within 100 steps” but it has become one of the hardest-hit areas by the COVID-19 [59]. This shows that the community residents network dominated by interest- and service-oriented social organizations cannot spontaneously transform into community governance capital and collective-action efficiency. The absence of political participation-oriented social organizations has left community residents in a state of “weak participation” and “atomized participation” in daily community affairs. Collective continuous participation based on the public interest of the community is rare. The consequence is that when a disaster strikes the community, residents lack the initiative to carry out collective actions spontaneously, and mutual trust and cooperation are not easy to establish due to lack of cooperation experience. Therefore, the effectiveness of collective action is low.

Secondly, COVID-19-susceptible communities have got insufficient governance resilience. The imperfect governance structure and the replacement of communities’ autonomy with administrative management are common features of most urban communities in China. It is common for old community residents’ committees to take charge of property management, while new commercial housing communities often lack the self-governing organization, namely a homeowners’ committee. An outstanding problem brought about by the governance structure is insufficient human resources for community governance. Once the community was hit by a critical disaster, the community’s emergency management personnel was overwhelmed. Moreover, the extension of the hierarchical administrative management style in communities not only leads to the weak self-government ability of the community residents, but also makes it difficult for the coordination of multiple governance bodies such as the grass-roots formal organizations (residents’ committees), informal organizations (homeowners’ committees, social organizations), market organizations (property management agencies), and residents. The hierarchical administrative relationship between residents’ committees and higher-level governments has also led to the lack of initiative and autonomy of the former. Thereby, in the process of disaster response, residents’ committees usually show behavioral inertia and passively execute orders and tasks of higher-level governments, cannot act decisively, autonomously, and respond flexibly in the face of a crisis. Lack of decision-making capacity and adaptability of policy implementation to local conditions constitute two important reasons why some communities become high-risk communities.

## Conclusion

Based on the assessment framework of community resilience, this study adopts a crisp-set qualitative comparative analysis method to examine the pandemic prevention and control capacity of 40 communities in Wuhan during the COVID-19 pandemic. Subsequently, the paper discusses the influencing variables and their configuration paths affecting the risk resilience of urban communities. The study found that vulnerable communities, alienated communities, and inefficient communities constitute three types of COVID-19-susceptible communities. Vulnerable communities inhabited by disadvantaged groups are not uniquely vulnerable to the pandemic as middle-class and wealthy communities can also be high-risk areas. The spatial dimension, rather than the capital dimension, constitutes the key influencing variable that affects the risk resilience of communities. Insufficient spatial resilience constitutes a necessary condition for COVID-19-susceptible communities, and its influence exceeds the human and economic capital dimension.

The contributions of this study are double: First, based on the QCA approach, we have identified the key influencing factors of pandemic-related community resilience, which is a missing element in the current literature; Second, through analyzing the consistency of seven condition variables, we discovered that the spatial resilience rather than capital or social resilience constituted the key factor of community resilience in the situation of a pandemic, finding constitutes a novelty compared to existing ideas which tend to emphasize the imbalanced importance of social network or socio-economic capital of the community. This study pointed out the main reason that communities populated by disadvantaged populations are vulnerable to COVID-19 might be the physical structures and environment of these communities usually lack spatial resilience, rather than their disadvantaged socio-economic capital.

Our findings suggest city policy-makers in new similar crises pay more attention to communities and places that lack spatial resilience, including identifying these places beforehand, taking appropriate risk precautions such as strengthening pandemic surveillance and prevention, taking locking-down measures in very early stage, transferring populations to places with strong spatial resilience if necessary. Meanwhile, this study confirmed the existing hypothesis that COVID-19-susceptible communities are the result of a comprehensive combination of weaknesses in multiple resilience dimensions. Empowering communities’ social capital and economic capital, as well as improving their governance capacity, constitute indispensable measures to improve a given pandemic susceptible community’s ability to respond to the disaster.

## Data Availability

The datasets used and/or analyzed during the current study are available from Wuhan Municipal Health Commission(http://wjw.wuhan.gov.cn/), China neighborhood website (http://www.cncn.org.cn/) and Message board for leaders (http://liuyan.people.com.cn/). The public access to the databases is open.
